# Denisovan and Neanderthal archaic introgression differentially impacted the genetics of complex traits in modern populations

**DOI:** 10.1186/s12915-022-01449-2

**Published:** 2022-11-07

**Authors:** Dora Koller, Frank R. Wendt, Gita A. Pathak, Antonella De Lillo, Flavio De Angelis, Brenda Cabrera-Mendoza, Serena Tucci, Renato Polimanti

**Affiliations:** 1 Department of Psychiatry, Yale University School of Medicine, West Haven, CT 06516 USA; 2 VA CT Healthcare Center, West Haven, CT 06516 USA; 3 Department of Genetics, Microbiology and Statistics, Faculty of Biology, University of Barcelona, Barcelona, Catalonia 08028 Spain; 4 Department of Biology, University of Rome “Tor Vergata”, Rome, 00133 Italy; 5 Department of Anthropology, Yale University, New Haven, CT 06511 USA

**Keywords:** Natural selection, Evolution, archaic introgression, Diversity, Heritability, Phenome

## Abstract

**Background:**

Introgression from extinct Neanderthal and Denisovan human species has been shown to contribute to the genetic pool of modern human populations and their phenotypic spectrum. Evidence of how Neanderthal introgression shaped the genetics of human traits and diseases has been extensively studied in populations of European descent, with signatures of admixture reported for instance in genes associated with pigmentation, immunity, and metabolic traits. However, limited information is currently available about the impact of archaic introgression on other ancestry groups. Additionally, to date, no study has been conducted with respect to the impact of Denisovan introgression on the health and disease of modern populations. Here, we compare the way evolutionary pressures shaped the genetics of complex traits in East Asian and European populations, and provide evidence of the impact of Denisovan introgression on the health of East Asian and Central/South Asian populations.

**Results:**

Leveraging genome-wide association statistics from the Biobank Japan and UK Biobank, we assessed whether Denisovan and Neanderthal introgression together with other evolutionary genomic signatures were enriched for the heritability of physiological and pathological conditions in populations of East Asian and European descent. In EAS, Denisovan-introgressed loci were enriched for coronary artery disease heritability (1.69-fold enrichment, *p*=0.003). No enrichment for archaic introgression was observed in EUR. We also performed a phenome-wide association study of Denisovan and Neanderthal alleles in six ancestry groups available in the UK Biobank. In EAS, the Denisovan-introgressed SNP rs62391664 in the major histocompatibility complex region was associated with albumin/globulin ratio (beta=−0.17, *p*=3.57×10^−7^). Neanderthal-introgressed alleles were associated with psychiatric and cognitive traits in EAS (e.g., “No Bipolar or Depression”-rs79043717 beta=−1.5, *p*=1.1×10^−7^), and with blood biomarkers (e.g., alkaline phosphatase-rs11244089 beta=0.1, *p*=3.69×10^−116^) and red hair color (rs60733936 beta=−0.86, *p*=4.49×10^−165^) in EUR. In the other ancestry groups, Neanderthal alleles were associated with several traits, also including the use of certain medications (e.g., Central/South East Asia: indapamide – rs732632 beta=−2.38, *p*=5.22×10^−7^).

**Conclusions:**

Our study provides novel evidence regarding the impact of archaic introgression on the genetics of complex traits in worldwide populations, highlighting the specific contribution of Denisovan introgression in EAS populations.

**Supplementary Information:**

The online version contains supplementary material available at 10.1186/s12915-022-01449-2.

## Background

Genetic variation across worldwide populations reflects the widespread impact of human evolutionary history, including processes related to natural selection and demographic history [1]. Large-scale genome-wide association studies (GWAS) are disentangling the complex genetic architecture of human traits and diseases, providing insights into the molecular and cellular mechanisms at the basis of physiological and pathological conditions [2–5]. Leveraging genome-wide data from these studies, it is possible to investigate whether the SNP-based heritability (SNP-*h*^*2*^, i.e., the proportion of phenotypic variance explained by additive effects of common genetic variation) of human phenotypes is enriched for specific genomic features via a partitioned heritability analysis [6]. Genomic features related to natural selection are enriched for loci associated with complex traits [1, 7–9]. In particular, background selection (i.e., the selective removal of deleterious alleles across the genome) appears to play a primary role in shaping the highly polygenic architecture of human traits and diseases [1, 7–9]. Positive selection, a measure for adaptive evolution was also detected in complex traits previously [10–12]. Introgression from Neanderthals and Denisovans, the only archaic humans sequenced to date, also contributes to the genetic pool of modern populations [13, 14] and consequently to the human phenotypic spectrum [15, 16]. The genomic segments of anatomically modern humans inherited from the admixture events with extinct human species are hypothesized to have contributed to the adaptation processes of worldwide populations that occurred during the colonization of landmasses [17–21]. Additionally, signatures of archaic introgression have been reported in genes associated with hair and skin pigmentation, immunity [16, 17, 19, 20, 22], neoplasms and metabolic traits [19, 23, 24], and male sterility [17, 18]. In populations of European descent (EUR), a phenome-wide association study of Neanderthal-introgressed alleles showed a wide range of associations with physiological conditions related to the immune system, skin pigmentation, and metabolic pathways, and with pathological outcomes such as depression, actinic keratosis, hypercoagulation, and tobacco use [15]. Due to the well-known disparities of ancestry representation in biomedical research, the information currently available regarding the role of human evolutionary history in shaping the genetic architecture of traits and diseases is mostly for EUR individuals. Although few studies investigated archaic introgression in non-EUR-descent groups such as Pacific [25], East Asian [26], Tibetan [27], and Island South East [28] populations, to our knowledge no investigation systematically explored the impact of archaic introgression across human traits and diseases across multiple ancestry groups. This major gap has important implications for the characterization of the history of human populations and its phenotypic consequences on individuals of diverse ancestral backgrounds.

The present study aimed to investigate how archaic introgressions contribute to the polygenic inheritance of human diseases and traits across different ancestry groups. Leveraging data generated from large-scale GWAS conducted in Biobank Japan (BBJ) [29, 30], we analyzed the genetic background of individuals of East Asian descent (EAS). Populations in East Asia present an evolutionary history that is only partially shared with EUR populations. With respect to archaic introgression, earlier studies found that on average, an EAS individual carries a higher percentage of Neanderthal genome DNA than a EUR individual (1.4% and 1.1%, respectively) [17]. EAS populations also show evidence of introgression from Denisovans [18, 19, 31]. Accordingly, we explored how archaic introgression and other evolutionary processes contributed to the genetics of complex traits in EAS and EUR populations. We also conducted a phenome-wide association study (PheWAS) of Neanderthal- and Denisovan-introgressed alleles to characterize their contribution in EAS and EUR individuals and other ancestry groups (CSA: Central/South Asian, MID: Middle Eastern, AMR: Admixed American) available from the UK Biobank (UKBB) [32]. We did not investigate data from UKBB participants of African descent, because no archaic introgression is present in these human groups. Similarly, Denisovan archaic introgression was investigated only in EAS and CSA. Neanderthal introgression was investigated in EAS, EUR, CSA, MID, and AMR. Our findings expand the understanding of how human evolutionary history influenced the genetic liability to complex traits, also providing evidence of the contribution of Denisovan introgression to physiological and pathological conditions in EAS populations.

## Results

### Partitioned heritability analysis

For the partitioned heritability analysis based on baseline and evolutionary annotations of the human genome, we identified a total of 37 and 39 traits with adequate SNP-*h*^*2*^ estimates (*z*-score ≥ 7) among those available in both BBJ (EAS participants) and UKBB (EUR participants), respectively (Additional file 1: Table S1). As expected, we observed a strong correlation between effective sample size and heritability *z*-score in both EAS and EUR (*ρ*=0.75, *p*=1.86×10^−13^ and *ρ*=0.82, *p*=2.20×10^−16^, respectively) (Additional file 1: Table S2). We identified several differences between EAS and EUR enrichments of genome structure and functional annotations (Additional file 1: Table S3). Although some of them may be due to the difference in the statistical power of UKBB and BBJ GWAS, we identified several enrichments that were statistically significant in EAS but not EUR.

We also observed several enrichments for evolutionary features in the SNP-*h*^*2*^ of traits and diseases assessed in EAS and EUR individuals (Table 1, Additional file 1: Table S4). With respect to archaic introgression, we identified one FDR-significant SNP-*h*^*2*^ enrichment: Denisovan-introgressed loci for coronary artery disease in EAS (1.7-fold enrichment, *p*=0.003). No enrichment for archaic introgression was observed in EUR (Supplemental Table 4). In line with previous studies [10, 33], the strongest enrichments in both ancestry groups were observed for annotations related to genic and LoF intolerant regions. In EAS, 89% and 68% of the traits had significant SNP-*h*^*2*^ enrichments for genic and LoF intolerant regions (False Discovery Rate, FDR *q*<0.05; Table 1). Platelet count was the most significantly enriched trait in both genic and LoF intolerant regions (1.33-fold enrichment, *p*=7.64×10^−12^ and 2-fold enrichment, *p*=1.98×10^−8^, respectively). Additionally, we identified several significant enrichments related to B-statistic values in EUR (i.e., reduction in allelic diversity due to purifying selection). Due to the much larger sample GWAS size, all phenotypes in EUR showed FDR significant enrichment in at least one of the B-statistic value thresholds. Background selection was more significantly enriched in lymphocyte count in EUR compared to EAS (EUR: 1.82-fold enrichment, *p*=1.12×10^−18^; EAS: 1.30-fold enrichment, *p*=2.18×10^−4^; EAS-EUR difference: *p*=4.59×10^−4^). Similar to other studies conducted in EUR [34, 35], we did not identify SNP-*h*^*2*^ enrichment for positive selection signatures in our EAS and EUR analyses (Additional file 1: Table S4).

**Table 1:** Statistically significant enrichment for natural selection and functional annotations in SNP-based heritability of complex traits in East Asian and European populations. Details of all traits and annotations tested are available in Supplemental Table 4

Trait	Annotation	East Asian			European			EAS-EUR Difference	
		Fold-enrichment	SE	***P***-value	Fold-enrichment	SE	***P***-value	***Z*** score	***P***-value
**Alanine aminotransferase**	B top1	0.855	0.349	0.983	2.728	0.614	0.005	−2.652	0.008
	B top2	−13.622	9.421	0.958	2.159	0.366	0.001	−1.674	0.094
	Genic	1.237	0.081	0.002	1.315	0.034	2.48E−18	−0.885	0.376
	LoF intolerant	1.272	0.232	0.227	1.615	0.121	2.18E−07	−1.311	0.190
**Albumin**	B top05	−1.971	2.266	0.718	3.950	1.484	0.049	−2.186	0.029
	B top1	1.135	0.675	0.983	3.158	0.947	0.024	−1.740	0.082
	B top2	8.223	7.181	0.958	3.734	0.670	6.11E−05	0.622	0.534
	Genic	1.380	0.092	1.82E−05	1.297	0.027	2.97E−19	0.866	0.386
	LoF intolerant	1.532	0.284	0.053	1.648	0.110	1.59E−09	−0.381	0.704
**Albumin/globulin ratio**	B top05	2.151	2.822	0.718	4.159	1.164	0.006	−0.658	0.511
	B top1	1.597	0.453	0.849	2.719	0.597	0.004	−1.498	0.134
	B top2	1.794	1.880	0.953	2.943	0.632	0.002	−0.579	0.563
	Genic	1.437	0.079	1.35E−09	1.310	0.030	2.07E−16	1.502	0.133
	LoF intolerant	2.056	0.283	5.44E−05	1.661	0.117	3.37E−09	1.294	0.196
**Aspartate aminotransferase**	B top05	−1.507	1.256	0.718	3.323	1.120	0.033	−2.870	0.004
	B top1	2.930	2.220	0.983	2.985	0.647	0.002	−0.024	0.981
	B top2	−12.232	6.942	0.958	2.742	0.496	0.000285	−2.152	0.031
	Genic	1.287	0.067	2.26E−06	1.314	0.030	2.39E−22	−0.371	0.711
	LoF intolerant	1.503	0.213	0.010	1.596	0.120	7.26E−08	−0.381	0.703
**Asthma**	B top05	−2.439	3.102	0.950	2.329	0.629	0.034	−1.506	0.132
	B top1	3.842	2.358	0.983	2.486	0.524	0.004	0.561	0.575
	B top2	−8.241	1.204	0.961	2.096	0.459	0.016	−8.020	1.06E−15
	Genic	1.008	0.085	0.928	1.123	0.040	0.001	−1.235	0.217
	LoF intolerant	0.869	0.236	0.575	1.223	0.095	0.016	−1.394	0.163
**Blood sugar**	B top2	−1.152	1.446	0.953	2.446	0.533	0.003	−2.334	0.020
	Genic	1.368	0.089	2.51E−05	1.293	0.065	2.09E−09	0.682	0.495
	LoF intolerant	1.324	0.274	0.217	1.635	0.192	2.06E−04	−0.930	0.352
**Blood urea nitrogen**	Genic	1.237	0.073	3.28E−04	NA	NA	NA	NA	NA
	LoF intolerant	1.471	0.238	0.043	NA	NA	NA	NA	NA
**Body mass index**	Genic	1.167	0.065	0.007	1.125	0.017	7.18E−12	0.628	0.530
	LoF intolerant	2.086	0.236	2.05E−07	1.682	0.069	1.46E−20	1.643	0.100
**Chloride**	Genic	1.361	0.105	3.80E−05	NA	NA	NA	NA	NA
	LoF intolerant	1.722	0.338	0.019	NA	NA	NA	NA	NA
**Coronary artery disease**	B top05	1.338	1.226	0.718	3.230	0.968	0.020	−1.211	0.226
	B top2	1.051	1.058	0.953	2.400	0.473	0.002	−1.164	0.244
	Denisovan	1.691	0.877	0.003	2.149	1.522	0.449	−0.261	0.794
	Genic	1.232	0.047	5.47E−08	1.250	0.040	1.48E−09	−0.282	0.778
	LoF intolerant	1.575	0.181	3.36E−04	1.681	0.141	3.07E−07	−0.460	0.645
**Diastolic blood pressure**	B top05	2.871	2.759	0.718	2.907	0.913	0.039	−0.012	0.990
	B top1	1.124	0.341	0.983	2.532	0.479	0.001	−2.394	0.017
	B top2	3.116	1.191	0.958	2.323	0.298	9.33E−06	0.646	0.519
	Genic	1.271	0.084	3.07E−04	1.230	0.024	5.69E−18	0.472	0.637
	LoF intolerant	2.050	0.288	5E−05	1.751	0.093	4.46E−15	0.988	0.323
**Estimated glomerular filtration rate**	B top05	1.234	1.484	0.718	4.724	1.209	0.002	−1.823	0.068
	B top1	2.184	0.471	0.983	3.960	0.848	4.97E−04	−1.830	0.067
	B top2	9.394	6.212	0.006	2.955	0.444	8.23E−06	1.034	0.301
	Genic	1.277	0.050	1.33E−08	1.306	0.031	2.23E−21	−0.483	0.629
	LoF intolerant	1.931	0.203	3.96E−07	1.853	0.135	3.09E−09	0.320	0.749
**Hematocrit**	Genic	1.342	0.072	1.45E−07	NA	NA	NA	NA	NA
	LoF intolerant	2.193	0.280	1.99E−06	NA	NA	NA	NA	NA
**Hemoglobin**	Genic	1.397	0.092	2.85E−07	NA	NA	NA	NA	NA
	LoF intolerant	2.336	0.342	4.79E−06	NA	NA	NA	NA	NA
**Lymphocyte count**	B top1	1.392	0.437	0.960	3.024	0.742	0.006	−1.896	0.058
	B top2	2.193	1.216	0.293	2.722	0.484	3.32E−04	−0.404	0.686
	Genic	1.178	0.079	0.015	1.316	0.028	2.70E−20	−1.636	0.102
	LoF intolerant	1.523	0.246	0.024	1.671	0.110	5.54E−09	−0.549	0.583
**Mean arterial pressure**	B top05	1.536	2.312	0.950	2.550	0.766	0.045	−0.416	0.677
	B top1	1.085	0.372	0.983	2.220	0.436	0.005	−1.980	0.048
	B top2	1.833	1.321	0.958	2.083	0.280	7.84E−05	−0.185	0.853
	Genic	1.314	0.076	6.07E−06	1.219	0.024	2.03E−16	1.189	0.234
	LoF intolerant	2.092	0.260	2.57E−06	1.784	0.096	7.51E−16	1.112	0.266
**Mean corpuscular hemoglobin**	B top05	3.948	6.980	0.718	4.182	1.496	0.030	−0.033	0.974
	B top1	2.015	0.836	0.849	3.802	1.055	0.007	−1.327	0.184
	B top2	2.520	2.993	0.953	4.056	0.734	1.64E−05	−0.498	0.618
	Genic	1.303	0.075	8.83E−06	1.348	0.046	1.19E−12	−0.515	0.607
	LoF intolerant	1.723	0.278	0.006	1.705	0.167	6.71E−06	0.056	0.955
**Mean corpuscular volume**	B top1	1.893	0.630	0.849	3.790	1.064	0.009	−1.534	0.125
	B top2	3.423	3.146	0.953	3.715	0.666	4.42E−05	−0.091	0.928
	Genic	1.311	0.061	2.22E−07	3.715	0.666	4.42E−05	−3.591	3.29E−04
	LoF intolerant	1.657	0.243	0.006	1.699	0.154	4.14E−06	−0.143	0.886
**Monocyte count**	B top05	1.967	2.920	0.718	5.248	2.118	0.045	−0.910	0.363
	B top1	2.456	1.347	0.849	3.793	1.004	0.005	−0.796	0.426
	B top2	1.348	0.975	0.013	2.773	0.628	0.005	−1.228	0.219
	Genic	1.255	0.086	0.002	1.318	0.040	7.53E−13	−0.660	0.509
	LoF intolerant	1.548	0.240	0.018	1.557	0.150	8.76E−05	−0.030	0.976
**Neutrophil count**	B top05	1.800	1.885	0.950	4.148	1.106	0.004	−1.074	0.283
	B top1	1.096	0.381	0.983	3.184	0.750	0.003	−2.483	0.013
	B top2	4.964	0.986	0.958	2.751	0.518	0.001	1.985	0.047
	Genic	1.235	0.080	0.002	1.256	0.025	3.62E−21	−0.248	0.804
	LoF intolerant	1.471	0.210	0.021	1.766	0.107	4.15E−11	−1.255	0.210
**Non-albumin protein**	B top05	4.440	5.075	0.718	4.291	1.014	0.001	0.029	0.977
	B top1	1.817	0.620	0.849	2.675	0.549	0.002	−1.036	0.300
	B top2	2.184	2.456	0.953	2.847	0.540	0.001	−0.264	0.792
	Genic	1.468	0.094	2.72E−09	1.315	0.033	6.72E−16	1.532	0.125
	LoF intolerant	2.373	0.301	9.41E−07	1.714	0.114	1.57E−10	2.043	0.041
**Platelet count**	B top1	3.575	0.375	0.983	4.781	1.276	0.004	−0.907	0.364
	B top2	2.644	0.641	0.958	3.744	0.586	6.10E−06	−1.267	0.205
	Genic	1.330	0.044	7.64E−12	1.366	0.026	7.71E−24	−0.710	0.478
	LoF intolerant	1.998	0.166	1.98E−08	1.984	0.142	5.76E−11	0.064	0.949
**Potassium**	B top1	1.112	0.363	0.983	2.387	0.390	3.54E−04	−2.393	0.017
	B top2	2.507	1.948	0.958	2.326	0.292	2.99E−06	0.092	0.927
	Genic	1.306	0.078	1.36E−05	1.103	0.032	0.002	2.425	0.015
	LoF intolerant	1.944	0.284	5.48E−05	1.641	0.115	7.51E−08	0.992	0.321
**Pulse pressure**	Genic	1.393	0.094	3.91E−07	NA	NA	NA	NA	NA
	LoF intolerant	2.304	0.306	2.14E−06	NA	NA	NA	NA	NA
**Red blood cell count**	B top05	1.639	2.885	0.718	4.790	1.325	0.004	−0.993	0.321
	B top1	2.173	0.691	0.849	3.476	0.724	0.001	−1.303	0.193
	B top2	5.614	1.771	0.958	3.033	0.487	1.83E−05	1.405	0.160
	Genic	1.353	0.066	4.87E−08	1.130	0.025	4.54E−07	3.159	0.002
	LoF intolerant	2.077	0.255	1.00E−05	1.561	0.092	1.19E−09	1.901	0.057
**Serum creatinine**	B top05	8.840	5.950	0.021	4.724	1.209	0.002	0.678	0.498
	B top1	3.121	1.461	0.983	3.960	0.848	4.97E−04	−0.497	0.619
	B top2	1.492	1.396	0.718	2.955	0.444	8.23E−06	−0.998	0.318
	Genic	1.289	0.052	7.98E−09	1.306	0.031	2.23E−21	−0.277	0.782
	LoF intolerant	1.865	0.194	9.85E−07	1.853	0.135	3.09E−09	0.050	0.960
**Smoking behaviors: Smoking initiation**	Genic	1.041	0.129	0.754	NA	NA	NA	NA	NA
	LoF intolerant	1.379	0.324	0.248	NA	NA	NA	NA	NA
**Sodium**	B top05	3.381	4.513	0.718	2.171	0.552	0.036	0.266	0.790
	B top1	1.362	0.715	0.983	1.953	0.329	0.003	−0.750	0.453
	B top2	−1.750	1.570	0.958	1.700	0.209	0.001	−2.178	0.029
	Genic	1.426	0.138	1.07E−04	1.130	0.025	4.54E−07	2.111	0.035
	LoF intolerant	2.193	0.485	0.006	1.561	0.092	1.19E−09	1.283	0.200
**Systolic blood pressure**	B top05	1.752	2.069	0.718	2.296	0.515	0.013	−0.255	0.799
	B top1	1.014	0.389	0.985	2.035	0.334	0.002	−1.991	0.047
	B top2	3.253	1.345	0.958	2.055	0.244	9.62E−06	0.876	0.381
	Genic	1.341	0.070	8.58E−08	1.234	0.024	3.92E−18	1.439	0.150
	LoF intolerant	2.143	0.241	8.95E−08	1.795	0.087	3.96E−18	1.356	0.175
**Total cholesterol**	B top1	4.831	2.371	0.983	4.722	1.431	0.008	0.039	0.969
	B top2	−7.322	9.190	0.953	2.873	0.781	0.012	−1.105	0.269
	Genic	1.359	0.086	2.71E−05	1.308	0.034	3.49E−13	0.553	0.580
	LoF intolerant	1.287	0.303	0.308	1.432	0.174	0.011	−0.414	0.679
**Total protein**	B top05	5.388	5.538	0.718	4.096	0.826	2.83E−04	0.231	0.818
	B top1	1.956	0.514	0.849	2.736	0.520	0.001	−1.066	0.286
	B top2	1.610	1.907	0.953	2.825	0.405	5.76E−06	−0.623	0.533
	Genic	1.395	0.093	2.96E−07	1.310	0.027	5.72E−18	0.874	0.382
	LoF intolerant	2.111	0.278	2.98E−05	1.745	0.129	1.75E−10	1.197	0.231
**Type 2 diabetes**	B top05	2.218	1.431	0.718	2.204	0.501	0.016	0.009	0.993
	B top1	1.085	0.292	0.983	2.109	0.392	0.005	−2.092	0.036
	B top2	5.468	3.974	0.293	2.090	0.254	6.08E−06	0.848	0.396
	Genic	1.171	0.034	8.13E−07	1.147	0.032	8.17E−06	0.523	0.601
	LoF intolerant	1.955	0.179	1.56E−07	1.608	0.123	2.03E−06	1.596	0.110
**White blood cell count**	B top05	5.024	3.190	0.718	5.029	1.350	0.003	−0.001	0.999
	B top1	1.200	0.354	0.983	3.307	0.666	0.001	−2.793	0.005
	B top2	−5.583	7.351	0.953	2.757	0.479	2.79E−04	−1.132	0.258
	Genic	1.340	0.073	5.24E−07	1.253	0.023	3.31E−23	1.148	0.251
	LoF intolerant	1.728	0.204	1.59E−04	1.730	0.100	8.96E−12	−0.007	0.995

The enrichment of three traits (i.e., blood sugar, mean corpuscular volume, non-albumin protein) was different for H3k27 active enhancer acetylation (H3K27ac) in EAS and EUR (most significant difference: non-albumin protein was more enriched for this functional annotation in EAS compared to EUR (EAS: 2.88-fold enrichment, *p*=1.22×10^−18^, EUR: 1.11-fold enrichment, *p*=0.080, EAS-EUR difference: *p*=2.96×10^−12^). Moreover, albumin/globulin ratio was depleted for H3K27ac flanking region in EAS (6.14-fold depletion, *p*=0.001), but it was significantly enriched in EUR (2.21-fold enrichment, *p*=2.26×10^−10^; EAS-EUR difference: *p*=2.72×10^−4^). The super-enhancer annotation was enriched in EAS (4.46-fold enrichment, *p*=3.01×10^−17^), but not in EUR (1.14-fold enrichment, *p*=0.105) with respect to non-albumin protein (EAS-EUR difference: *p*=3.43×10^−12^). The enrichment of three traits (i.e., lymphocyte count, neutrophil count, non-albumin protein) was different for CpG content between EAS and EUR. The most significant difference was for non-albumin protein, which was more enriched for this functional annotation in EAS compared to EUR (EAS: 1.51-fold enrichment, *p*=1.37×10^−11^, EUR: 1.09-fold enrichment, *p*=2.36×10^−6^, EAS-EUR difference: 2.89×10^−6^).

### Phenome-wide association study of Archaic introgressed loci

Although we observed only SNP-*h*^*2*^ enrichment with respect to Denisovan-introgressed loci for coronary artery disease in EAS, single loci inherited from Neanderthals and Denisovans can still contribute to the phenotypic variation of human populations [15]. Therefore, we performed a PheWAS of Neanderthal and Denisovan introgressed loci across multiple ancestry groups available from the UK Biobank (Additional file 1: Table S5) and identified several associations surviving FDR multiple testing correction at 1%. In our analysis, we tested introgressed loci that (i) matched only Neanderthal genome, (ii) matched only Denisovan genome, and (iii) matched both Neanderthal and Denisovan genomes.

**Fig. 1:**
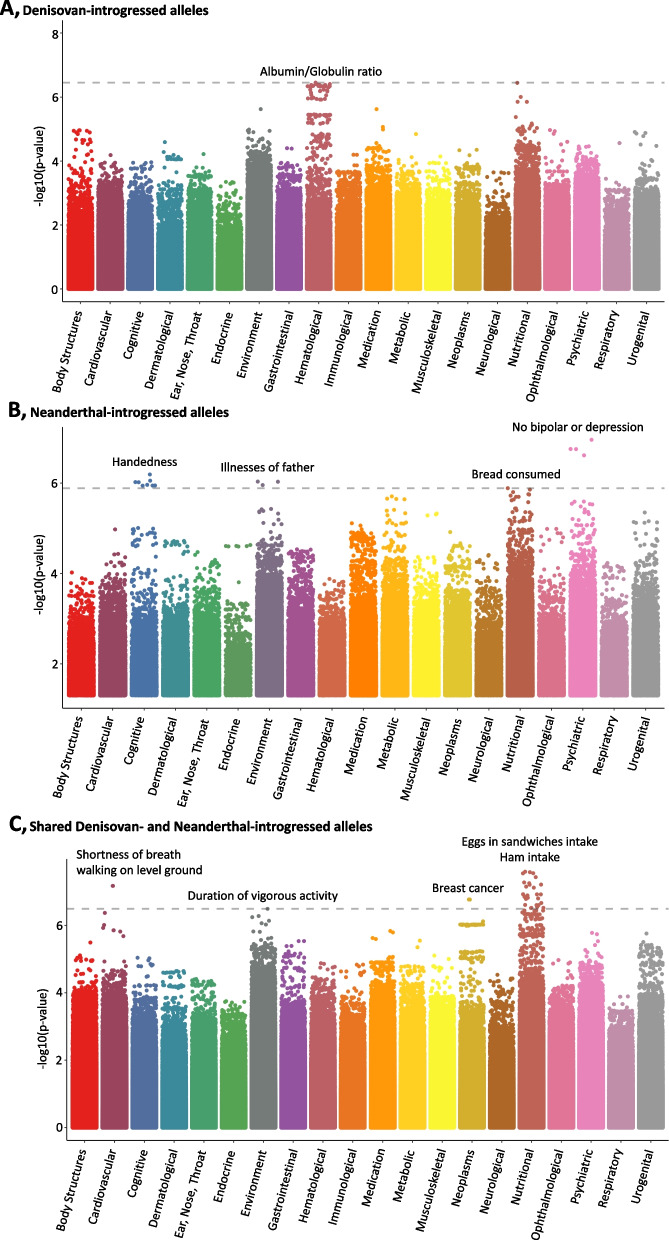
PheWAS Manhattan plots showing variant associations with UKBB phenotypes in EAS. Panel **A** shows associations with Denisovan-introgressed alleles, panel **B** depicts associations with Neanderthal-introgressed alleles, and panel **C** shows associations with introgressed alleles matching both Denisovan and Neanderthal genomes. Phenotype categories are shown on the *x*-axis, while -log10 (*p*-values) are shown on the *y*-axis. The dashed line shows the FDR-significant threshold (*q* < 0.01)

In EAS, Denisovan introgressed SNP rs62391664 was associated with albumin/globulin ratio (beta=−0.17, *p*=3.57×10^−7^; Fig. 1A, Additional file 1: Table S6). Among Neanderthal introgressed loci, rs79043717*A, rs145929965*C, and rs76966342*A alleles showed the strongest associations with respect to lower risk for “No bipolar or depression” (beta=−1.50, *p*=1.10×10^−7^), “handedness” (beta=−3.54, *p*=6.45×10^−7^), and “illnesses of father” (beta=−0.44, *p*=9.27×10^−7^), respectively (Fig. 1B, Additional file 1: Table S7). Introgressed alleles matching both Denisovan and Neanderthal genomes were associated with increased risk of “shortness of breath” (rs77589994*A beta=5.27, *p*=1.10×10^−8^) and “breast cancer” (rs12143332*A beta=1.56, *p*=1.69×10^−7^), and lower chance of “duration of vigorous activity” (rs74962884*G beta=-0.26, *p*=3.20×10^−7^, Fig. 1C, Additional file 1: Table S8). Among Neanderthal and Neanderthal/Denisovan introgressed loci, we also observed few associations related to dietary habits (e.g., “bread consumed”; Additional file 1: Tables S7 and S8).

In EUR, Neanderthal-introgressed alleles were associated with 82 phenotypes, being red hair color (rs60733936*A beta=−0.86, *p*=1.81×10^−157^) and alkaline phosphatase (rs11244089*A beta=−0.10, *p*=1.44×10^−109^), the most significant (Additional file 1: Table S9). Because of the large number of EUR-Neanderthal associations surviving multiple testing correction (FDR *q*<0.01), we tested whether these associations were specifically enriched for one or more of the phenotypic domains investigated (Additional file 1: Table S5), observing an over-representation of EUR-Neanderthal associations with metabolic traits (27.52-fold enrichment, *p*=6.61×10^−7^). Although a limited sample size is available for other ancestry groups available from UKBB, we identified several associations with Neanderthal-introgressed alleles in CSA and MID (Additional file 1: Tables S10–S11). No Denisovan- or Denisovan/Neanderthal-introgressed locus was associated to any phenotype in CSA (Additional file 1: Tables S12 and S13). Interestingly, some of the associations were related to the use of certain medications, including those related to pain management (e.g., aspirin in CSA) and opioids (MID) and antihypertensive medication (indapamide and alfuzosin in CSA). No association survived multiple testing correction in AMR (Additional file 1: Table S14).

### Enrichment for biological processes, cellular components, and molecular functions

Considering the loci identified in our PheWAS, we tested the enrichment for biological processes, cellular components, and molecular functions. Considering the Neanderthal loci identified in the PheWAS in EUR, we found 30 gene-set enrichments (FDR < 5%) related to genomic regulation (Additional file 1: Table S15). Among them, we observed genes targeted by several microRNAs (miRNA, e.g., Hsa-miR-374b, FDR *q*=9.27×10^−5^) and by different transcription factors (e.g., WT1 in human podocyte, FDR *q*=9.27×10^−9^). Due to the limited number of loci identified in other ancestries, no enrichment survived multiple testing correction.

## Discussion

Previous studies showed that Neanderthal-introgressed loci are associated with immunological, neurological, psychiatric, metabolic, cardiovascular, and dermatological outcomes in EUR populations [10, 15–17]. In our study, we expanded this previous knowledge by testing for enrichment and depletion of SNP-*h*^*2*^ for loci related to Denisovan- and Neanderthal-introgression and several other evolutionary features across multiple traits in EAS and EUR populations. Additionally, we provide the first evidence of the consequences of Denisovan introgression across the human phenotypic spectrum in human groups of East Asian descent.

Leveraging EAS genome-wide information, we observed that Denisovan-introgressed loci are more enriched with the heritability of coronary artery disease than expected by chance. Two related cardiovascular phenotypes, myocardial infarction, and coronary atherosclerosis were previously associated with Neanderthal-introgressed loci in EUR [15]. In our EAS PheWAS of introgressed loci matching Denisovan/Neanderthal loci, we identified an association with “shortness of breath walking on level ground”, which is a trait related to cardiovascular health [36]. The associated variants locus, rs77589994 mapped to the *TRAP1* gene that encodes a protein regulating cellular stress responses [37]. The first PheWAS of Neanderthal-introgressed alleles in EUR found that Neanderthal alleles explained a significant proportion of variance in risk in coronary atherosclerosis [15]. In line with this previous evidence, we observed that “vascular/heart problems diagnosed by doctor” was associated with a Denisovan-Neanderthal introgressed SNP, the *LINGO2* rs74597612 variant in EUR.

Our EUR PheWAS of Neanderthal-introgressed SNPs was enriched for associations related to metabolic traits. This overrepresentation was not present in the previous Neanderthal-introgression PheWAS. This could be due to the different characteristics of the cohorts investigated. Our PheWAS conducted in the UKBB, which is a middle-aged sample healthier and wealthier than the general population [38]. Conversely, the previous PheWAS was conducted in the Electronic Medical Records and Genomics (eMERGE) Network [39] which is a sample combining participants enrolled from multiple healthcare systems. Sample-specific characteristics may have affected the statistical power of detecting associations with respect to certain health domains. Similarly. another study investigating Neanderthal-introgressed SNPs in EAS and EUR found multiple associations with autoimmune diseases, prostate cancer, and type 2 diabetes [24]. This study used a different method to assign Neanderthal-introgressed alleles than the one applied in our study. In this previous analysis, Neanderthal-introgressed alleles were defined as those present in modern non-African populations that are shared with the Vindija Neandertal genome using a linkage disequilibrium-based test for incomplete lineage sorting (ILS). Considering loci identified from multiple sources, this previous investigation tested whether they were Neanderthal introgressed using the ILS method. Therefore, due to the different study designs, a different set of associations were identified. Another recent study investigating Neanderthal-introgressed alleles showed associations with hair color and hematological biomarkers that are consistent with our results [40].

In EAS, a Denisovan-introgressed allele was associated with a metabolic phenotype, albumin/globulin ratio. To our knowledge, this is the first evidence of the effect of Denisovan introgression on the phenotypic expression of EAS modern populations. In our EUR PheWAS, a Neanderthal-introgressed allele was associated with albumin. Although these are two different hematological parameters, the convergence on albumin-related biomarkers may suggest an evolutive pressure on archaic introgression with respect to genes involved in albumin regulation. Among Neanderthal introgressed alleles in EAS, we also identified an association with handedness. This trait is particularly interesting with respect to human evolution, because it arose after the chimp and human lines were separated between 5 and 7 million years ago [41]. Neanderthal hominins appear to be right-handed in line with manual lateralization and brain functional asymmetry that is also present in modern humans [42]. Accordingly, the association of Neanderthal-introgressed loci with handedness in EAS may suggest that of the human populations. With respect to pathological conditions, breast cancer was also associated with a shared Denisovan- and Neanderthal-introgressed variant in EAS. Although this is a novel finding, Neanderthal-introgressed haplotypes were previously associated with prostate cancer [24]. This may suggest that variants introgressed from archaic genomes may play a role in the pathogenesis of cancers linked to sex hormone regulation [43].

In our evolution-focused SNP-*h*^*2*^ enrichment analysis, we detected an overabundance of genic and LoF intolerant loci in both EAS and EUR, suggesting that functionally important regions of the genome contribute to SNP-*h*^*2*^ to a different extent compared to the other annotations tested [10, 33, 40, 44]. Most of the traits tested were also enriched in CpG content, which is known to be positively correlated with genic content [45]. Genic and LoF intolerant regions are strongly under negative selection [46]. While most EUR phenotypes (76%) were highly enriched in B-statistic values, we only found one FDR-significant association in EAS (serum creatinine). A similar disparity between EUR and EAS findings was also present for the B-statistic continuous annotation. This is likely due to the much larger sample size available in EUR and may not reflect a general lack of evidence for background selection in EAS populations (Supplemental Table 2). Conversely, some functional enrichments were significantly more enriched in EAS than in EUR. For example, the super-enhancer annotation was enriched in EAS, but not in EUR. Genomic regions including enhancers have been shown to present an accelerated evolutionary rate, which is a signature of positive selection [11]. However, similar to previous studies [34, 35], none of the positive-selection annotations tested was significant in the two populations tested. We also observed that several Neanderthal-introgressed loci identified were related to transcriptomic regulation via transcription factors (i.e., proteins that control transcription from DNA to mRNA) and miRNA (i.e., non-coding RNA responsible for RNA silencing and post-transcriptional gene expression regulation) in EUR. MiRNA seed regions are under significant background selection [47] and their function can be affected by variants introgressed from Neanderthals [48].

Although our study provides novel insights into the role of human evolutionary history in the genetics of traits and diseases in worldwide populations, we acknowledge that the results generated are strongly affected by the well-known overrepresentation of EUR populations in human genetic research [49]. Accordingly, the analyses conducted were more statistically more powerful when conducted in EUR-based datasets than in EAS-based ones. However, we demonstrated that the majority of functional annotations were not statistically different between EAS and EUR in their enrichment for the SNP-based heritability of complex traits. When a stronger enrichment was observed in EUR, we cannot distinguish whether this is due to the larger sample size available in EUR or to genetic differences between the two populations investigated. Conversely, when a stronger enrichment was observed in EAS, this is related to human genetic diversity. Nevertheless, it is important to highlight that our findings are consistent with the fact that the fundamental biology of human traits and disease is shared among worldwide populations and that diversity among ancestral groups affects only a relatively small component of the genetic predisposition to complex traits. Additionally, further studies are needed to disentangle the role of environment, demography, and natural selection in the inter-population differences observed.

## Conclusions

Our study expands the understanding of how archaic introgression contributed to the genetic architecture of human traits and diseases across worldwide populations. In particular, we present evidence that Denisovan and Neanderthal introgression contributed specifically to shape the genetics of complex traits in East Asian populations and other human groups currently underrepresented in genetic research. This highlights the need to expand the representation of human diversity in genetic research to ensure a comprehensive understanding of the complex dynamics by which the variation in the human genome is linked to the variation in the human phenome.

## Methods

### Datasets

GWAS statistics were accessed from BBJ [29, 30] and the UKBB [50]. BBJ is a registry of over 200,000 Japanese patients including information about 47 diseases and 59 quantitative traits (Supplemental Table 1) [29, 30]. The UKBB dataset provides information regarding more than 7000 phenotypes assessed in up to 500,000 participants from six ancestry groups [32]. We obtained genome-wide association statistics from a pan-ancestry genetic analysis of the UKBB (Pan-UKBB). A detailed description of this analysis is available at https://pan.ukbb.broadinstitute.org. Briefly, multi-ancestry genome-wide association analyses of 7,221 phenotypes were performed using a generalized mixed model association testing framework. We used ancestry-specific GWAS statistics available for five genetically-defined ancestry groups: EUR (*N*=420,531), CSA (*N*=8876), EAS (*N*=2709), MID (*N*=1599), AMR (*N*=980). We did not investigate data from UKBB participants of African descent, because no archaic introgression is present in these human groups. Similarly, Denisovan archaic introgression was investigated only in EAS and CSA. Neanderthal introgression was investigated in EUR, CSA, EAS, MID, and AMR.

### Annotations measuring archaic introgression, positive-and negative selection, and functionally important regions

SNP-*h*^*2*^ partitioning [6] was performed considering 95 baseline genomic annotations (baseline-LD model v2.2 downloaded from https://alkesgroup.broadinstitute.org/LDSCORE/) characterizing important molecular properties such as allele frequency distributions, conserved regions of the genome, and regulatory elements [9]. The full model included annotations from Finucane et. al. (2015) [6] including coding, UTR, promoter, and intronic regions. Then additional annotations were added to the model including four human promoter annotations (promoter, promoter from the Exome Aggregation Consortium [33], and two corresponding flanking annotations) [34], three human enhancer annotations (enhancer and corresponding flanking annotation + enhancer-enhancer conservation count) [34], two human promoter sequence age annotations (including one flanking annotation) [35], and two human enhancer sequence age annotation (including one flanking annotation) [35].

We created additional genome-wide annotations for Denisovan [51, 52] and Neanderthal [18, 51–53]-introgressed, positively selected [12, 35, 54], negatively selected [1, 55], genic and LoF intolerant [33] positions using the publicly available datasets from the original publications. Denisovan (*N*=6515) and Altai Neanderthal-introgressed (*N*=49,793) positions were derived from the Sprime dataset [52], which identified these archaic-introgressed positions from the 1000 Genome Project with respect to the Japanese population sample (i.e., Japanese in Tokyo, Japan). This reference population was selected because our primary analysis was conducted with respect to East Asian populations available from BBJ and UKB. We defined Denisovan SNPs as those matching the Denisovan genome. Neanderthal SNPs we selected were matched uniquely to the Neanderthal genome. The contribution of archaic ancestry was also assessed by another method that identifies segments of archaic ancestry in modern human genomes without the need for archaic reference genomes [18, 53].

Positive selection was tested based on the integrated haplotype score (iHS) for Asian populations, which reports detection of positive selection during the last ~30,000 years based on the detection of abnormally long haplotypes [56]. Cross-population extended haplotype homozygosity (XP-EHH) comparing EAS and EUR ancestries based on 1000 Genomes was also used to detect differential selective pressure since the two populations diverged [35]. The B-statistic for EAS was used to assess background selection. B measures phylogenetic information from other primates to determine the reduction in allelic diversity in humans due to purifying selection [1]. The Exome Aggregation Consortium (ExAC) database was used to annotate genic and LoF intolerant regions of the genome. Each gene was assigned a probability of LoF intolerance (pLI) score [33]. Continuous evolutionary measurements were analyzed as top 2%, top 1%, and top 0.5% of scores genome-wide as binary annotations as recommended before due to the difficulty of setting specific thresholds to define regions under negative- and positive selection [10, 44, 55]. The evolutionary annotations used in EUR are reported in Wendt et al. [10]. Apart from those previously reported, we created additional annotations for Denisovan- and Neanderthal-introgressed positions for EUR as explained before.

### Statistical analysis

#### Linkage Disequilibrium Score Regression

The Linkage Disequilibrium Score Regression method (LDSC) was used to quantify the enrichment of evolutionary annotations in the SNP-*h*^*2*^ of each trait [5]. For each binary trait, the effective sample size was calculated as recommended previously [57]. The major histocompatibility complex region was excluded from the analysis due to its complex LD structure. To compare BBJ EAS participants with other ancestry groups, we selected 79 UKBB traits that were assessed similarly to those available in BBJ. SNP-*h*^*2*^ was calculated for each phenotype and, as recommended by the developers [6], the 69 traits with an estimated SNP-*h*^*2*^*z* score ≥ 7 were selected for the partitioned SNP-*h*^*2*^ analysis to test whether certain functional categories of the human genome contribute disproportionately to the heritability of the traits investigated. Due to the limited sample size in UKBB for other ancestry groups, we limited our partitioned SNP-*h*^*2*^ analysis to the data derived from BBJ EAS and UKBB EUR participants. Accordingly, we used LD scores generated from the 1000 Genome Project Phase 3 EAS and EUR reference panels to analyze GWAS data generated from BBJ and UKBB, respectively [58]. We applied FDR multiple-testing correction (*q* ≤ 0.05) [59] accounting for the number of phenotypes tested. Partitioned SNP-*h*^*2*^ in LDSC analyzes a large linear model including all annotations described in the previous section simultaneously such that enrichment values for a single annotation reflect independence from all other annotations in the model.

#### Phenome-wide association study

To increase the resolution of our investigation (from heritability enrichment to single-variant contribution), we conducted a PheWAS of Denisovan (*N*=6515) and Neanderthal introgressed (*N*=49,793) loci, and shared loci between Denisovan-and Neanderthal (*N*=22,787) in EAS and CSA. As mentioned above, we only tested Neanderthal introgression in the other ancestry groups (AMR, EUR, MID). PheWAS tests for association between single variants and a large number of different phenotypes. The association statistics of 7,221 phenotypes were derived from the Pan-UKBB analysis (details available at https://pan.ukbb.broadinstitute.org, Additional file 1: Table S5). Briefly, the genome-wide association analysis was conducted using the Scalable and Accurate Implementation of Generalized (SAIGE) mixed model and including a kinship matrix as a random effect and covariates as fixed effects. The covariates included age, sex, age × sex, age^2^, age^2^×sex, and the top 10 within-ancestry principal components.

Our phenome-wide analysis included traits related to body structures, cardiovascular, cognitive, dermatological, ear-nose-throat, endocrine, environmental, gastrointestinal, hematological, immunological, medication, metabolic, musculoskeletal, neoplasms, neurological, nutritional, ophthalmological, psychiatric, respiratory, and urogenital domains (Supplemental Table 5). These phenotypic categories are similar to the ones used in the GWAS Atlas [60]. To keep the type I error rate at 1%, we applied FDR multiple testing correction considering *q* < 0.01 [59] accounting for the number of phenotypes, variants, and ancestries tested to identify associations surviving multiple testing correction. Variants with minor allele frequency (MAF) ≤ 0.05 and the variants with the “low-confidence” flag (i.e., variants with alternate allele count in cases ≤ 3, alternate allele count in controls ≤ 3, or minor allele count (cases and controls combined) ≤ 20) in the Pan UKBB analysis were excluded from the analysis. To define independent loci among those identified as significant by our PheWAS, we performed LD clumping using PLINK 1.9 [61] with a *r*^2^=0.1 within 500 kb windows. The significant LD-independent variants were annotated to genes using the SNP Nexus variant annotation tool [62].

#### Gene Ontology Enrichment

The significant genes identified in each PheWAS were analyzed for gene ontology enrichment using the ShinyGO toolset [63] using all protein-coding genes in the genome as background set and functional and molecular annotations (e.g., molecular pathways and gene ontology) from Ensembl [64]. Gene ontology enrichment is used to interpret sets of genes using Gene Ontology system [65] of classification based on their functional characteristics. We considered FDR *q* < 0.05 to identify enrichments surviving multiple testing correction.

#### Over-representation test

To test for over-representation of certain phenotypic classes among the associations observed in the PheWAS, we calculated the significance of the phenotypic enrichment by a hypergeometric distribution test (https://systems.crump.ucla.edu/hypergeometric/) where *k* is the number of phenotypes with at least one LD-independent association within the phenotype category of interest, *s* is the number of phenotypes with at least one LD-independent association, *M* is the number of phenotypes within the phenotype category of interest, and *N* is the number of phenotypes tested.

## Supplementary Information

**Additional file 1: Table S1.** Description of all phenotypes derived from each GWAS of East Asian and European individuals. **Table S2.** Comparison of the equivalent phenotypes derived from each GWAS of East Asian and European individuals. **Table S3.** Description of all phenotypes and statistics for baseline annotations derived from each GWAS of East Asian and European individuals. **Table S4.** Description of all phenotypes and statistics for evolutionary annotations derived from each GWAS of East Asian and European individuals with heritability z>7. Nominally significant enrichments (*p* < 0.05) are provided and FDR significant (*q* < 0.05) results are highlighted. **Table S5.** Traits from the UK Biobank included in the Phenome-Wide Association Study. The number of cases and controls and trait description are shown. **Table S6.** Significant association of Denisovan-introgressed variants with phenotypic traits from the Pan UKB in EAS. Beta value, SE, *p*-value, FDR q-value, gene annotation, predicted function, MAF, *p* value heterogeneity and q value heterogeneity are also reported. **Table S7.** Significant association of Neanderthal-introgressed variants with phenotypic traits from the Pan UKB in EAS. Beta value, SE, *p*-value, FDR q-value, gene annotation, predicted function, MAF, *p* value heterogeneity and q value heterogeneity are also reported. **Table S8.** Significant association of shared Denisovan- and Neanderthal-introgressed variants with phenotypic traits from the Pan UKB in EAS. Beta value, SE, *p*-value, FDR q-value, gene annotation, predicted function, MAF, *p* value heterogeneity and q value heterogeneity are also reported. **Table S9.** Significant association of Neanderthal-introgressed variants with phenotypic traits from the Pan UKB in EUR. Beta value, SE, *p*-value, FDR q-value, gene annotation, predicted function, MAF, *p* value heterogeneity and q value heterogeneity are also reported. **Table S10.** Significant association of Neanderthal-introgressed variants with phenotypic traits from the Pan UKB in CSA. Beta value, SE, *p*-value, FDR q-value, gene annotation, predicted function, MAF, *p* value heterogeneity and q value heterogeneity are also reported. **Table S11.** Significant association of Neanderthal-introgressed variants with phenotypic traits from the Pan UKB in MID. Beta value, SE, *p*-value, FDR q-value, gene annotation, predicted function, MAF, *p* value heterogeneity and q value heterogeneity are also reported. **Table S12.** Significant association of Denisovan-introgressed variants with phenotypic traits from the Pan UKB in CSA. Beta value, SE, *p*-value, FDR q-value, gene annotation, predicted function, MAF, *p* value heterogeneity and q value heterogeneity are also reported. **Table S13.** Significant association of shared Denisovan- and Neanderthal-introgressed variants with phenotypic traits from the Pan UKB in CSA. Beta value, SE, *p*-value, FDR q-value, gene annotation, predicted function, MAF, *p* value heterogeneity and q value heterogeneity are also reported. **Table S14.** Significant association of Neanderthal-introgressed variants with phenotypic traits from the Pan UKB in AMR. Beta value, SE, *p*-value, FDR q-value, gene annotation, predicted function, MAF, *p* value heterogeneity and q value heterogeneity are also reported. **Table S15.** Significant (FDR < 0.05) gene-set enrichments in the EUR PheWAS with Neanderthal-introgressed loci.

## Data Availability

All data generated or analyzed during this study are included in this published article, its supplementary information files, and publicly available repositories. All original code is deposited at the following repositories and is publicly available as of the date of publication. This paper does not report new code. Data used in this study are publicly available as of the date of publication. Biobank Japan summary statistics (2021, doi: 10.1038/s41588-021-00931-x): http://jenger.riken.jp/en/result. Pan-UKBB summary statistics (2022): https://pan.ukbb.broadinstitute.org/downloads. Baseline genomic annotations: https://alkesgroup.broadinstitute.org/LDSCORE/. Integrated haplotype score (iHS) (2006): http://hgdp.uchicago.edu/Browser_tracks/iHS/. Cross-population extended haplotype homozygosity (XP-EHH) (2007): http://hgdp.uchicago.edu/Browser_tracks/XPEHH/. B-statistic (2019): https://github.com/gmcvicker/bkgd/tree/7ae49926008406bfcc81aec419e5d314390338e1. Denisovan and Neanderthal positions (2018, doi: 10.17632/y7hyt83vxr.1): https://data.mendeley.com/datasets/y7hyt83vxr/1. Neanderthal local ancestry (2019): https://reich.hms.harvard.edu/datasets/landscape-neandertal-ancestry-present-day-humans. ExAC database (2016): https://gnomad.broadinstitute.org/. LDSC heritability and partitioned heritability (2015): https://github.com/bulik/ldsc/wiki.

## Abbreviations

AMR: Individuals of Admixed American ancestry
BBJ: Biobank Japan
CSA: Individuals of Central/South-Asian ancestry
EAS: Individuals of East Asian descent
eMERGE: Electronic Medical Records and Genomics
EUR: Individuals of European descent
ExAC: Exome Aggregation Consortium
FDR: False Discovery Rate
GWAS: Genome-wide association studies
ILS: Incomplete lineage sorting
LDSC: Linkage Disequilibrium Score Regression
LoF: Loss of function
MID: Individuals of Middle Eastern ancestry
miRNA: Micro ribonucleic acid
Pan-UKBB: Pan-ancestry genetic analysis of the UK Biobank
PheWAS: Phenome-wide association study
pLI: Probability of loss of function intolerance
SNP-*h*^*2*^: SNP-based heritability
UKBB: UK Biobank
XP-EHH: Cross-population extended haplotype homozygosity

## References

[1] McVicker G, Gordon D, Davis C, Green P (2009). Widespread genomic signatures of natural selection in hominid evolution. Plos Genet.

[2] Purcell SM, Wray NR, Stone JL, Visscher PM, O’Donovan MC, International Schizophrenia Consortium (2009). Common polygenic variation contributes to risk of schizophrenia and bipolar disorder. Nature.

[3] Yang J, Benyamin B, McEvoy BP, Gordon S, Henders AK, Nyholt DR (2010). Common SNPs explain a large proportion of the heritability for human height. Nat Genet.

[4] Stahl EA, Wegmann D, Trynka G, Gutierrez-Achury J, Diabetes Genetics Replication and Meta-analysis Consortium, Myocardial Infarction Genetics Consortium (2012). Bayesian inference analyses of the polygenic architecture of rheumatoid arthritis. Nat Genet.

[5] Bulik-Sullivan BK, Loh P-R, Finucane HK, Ripke S, Yang J, Schizophrenia Working Group of the Psychiatric Genomics Consortium (2015). LD Score regression distinguishes confounding from polygenicity in genome-wide association studies. Nat Genet.

[6] Finucane HK, Bulik-Sullivan B, Gusev A, Trynka G, Reshef Y, Loh P-R (2015). Partitioning heritability by functional annotation using genome-wide association summary statistics. Nat Genet..

[7] Zeng J, de Vlaming R, Wu Y, Robinson MR, Lloyd-Jones LR, Yengo L (2018). Signatures of negative selection in the genetic architecture of human complex traits. Nat Genet.

[8] Zeng J, Xue A, Jiang L, Lloyd-Jones LR, Wu Y, Wang H (2021). Widespread signatures of natural selection across human complex traits and functional genomic categories. Nat Commun.

[9] Gazal S, Finucane HK, Furlotte NA, Loh P-R, Palamara PF, Liu X (2017). Linkage disequilibrium-dependent architecture of human complex traits shows action of negative selection. Nat Genet.

[10] Wendt FR, Pathak GA, Overstreet C, Tylee DS, Gelernter J, Atkinson EG (2021). Characterizing the effect of background selection on the polygenicity of brain-related traits. Genomics.

[11] Moon JM, Capra JA, Abbot P, Rokas A (2019). Signatures of recent positive selection in enhancers across 41 human tissues. G3 (Bethesda).

[12] Grossman SR, Shlyakhter I, Shylakhter I, Karlsson EK, Byrne EH, Morales S (2010). A composite of multiple signals distinguishes causal variants in regions of positive selection. Science.

[13] Meyer M, Kircher M, Gansauge M-T, Li H, Racimo F, Mallick S (2012). A high-coverage genome sequence from an archaic denisovan individual. Science.

[14] Prüfer K, Racimo F, Patterson N, Jay F, Sankararaman S, Sawyer S (2014). The complete genome sequence of a Neanderthal from the Altai Mountains. Nature.

[15] Simonti CN, Vernot B, Bastarache L, Bottinger E, Carrell DS, Chisholm RL (2016). The phenotypic legacy of admixture between modern humans and Neandertals. Science.

[16] Dannemann M, Kelso J (2017). The contribution of Neanderthals to phenotypic variation in modern humans. Am J Human Genet.

[17] Sankararaman S, Mallick S, Dannemann M, Prüfer K, Kelso J, Pääbo S (2014). The genomic landscape of Neanderthal ancestry in present-day humans. Nature.

[18] Sankararaman S, Mallick S, Patterson N, Reich D (2016). The combined landscape of denisovan and neanderthal ancestry in present-day humans. Curr Biol.

[19] Vernot B, Tucci S, Kelso J, Schraiber JG, Wolf AB, Gittelman RM (2016). Excavating Neandertal and Denisovan DNA from the genomes of Melanesian individuals. Science.

[20] Vernot B, Akey JM (2014). Resurrecting surviving neandertal lineages from modern human genomes. Science.

[21] Gittelman RM, Schraiber JG, Vernot B, Mikacenic C, Wurfel MM, Akey JM (2016). Archaic hominin admixture facilitated adaptation to out-of-Africa environments. Curr Biol.

[22] McArthur E, Rinker D, Capra JA. Quantifying the contribution of Neanderthal introgression to the heritability of complex traits. Nat Commun. 2021;12:4481. 10.1038/s41467-021-24582-y PMC8298587 34294692

[23] Skov L, Coll Macià M, Sveinbjörnsson G, Mafessoni F, Lucotte EA, Einarsdóttir MS (2020). The nature of Neanderthal introgression revealed by 27,566 Icelandic genomes. Nature.

[24] Dannemann M (2021). The population-specific impact of Neandertal introgression on human disease. Genome Biol Evol.

[25] Choin J, Mendoza-Revilla J, Arauna LR, Cuadros-Espinoza S, Cassar O, Larena M (2021). Genomic insights into population history and biological adaptation in Oceania. Nature.

[26] Taskent O, Lin YL, Patramanis I, Pavlidis P, Gokcumen O (2020). Analysis of haplotypic variation and deletion polymorphisms point to multiple archaic introgression events, including from Altai Neanderthal lineage. Genetics.

[27] Huerta-Sánchez E, Jin X, Null A, Bianba Z, Peter BM, Vinckenbosch N (2014). Altitude adaptation in Tibetans caused by introgression of Denisovan-like DNA. Nature.

[28] Teixeira JC, Jacobs GS, Stringer C, Tuke J, Hudjashov G, Purnomo GA (2021). Widespread Denisovan ancestry in Island Southeast Asia but no evidence of substantial super-archaic hominin admixture. Nat Ecol Evol.

[29] Kanai M, Akiyama M, Takahashi A, Matoba N, Momozawa Y, Ikeda M (2018). Genetic analysis of quantitative traits in the Japanese population links cell types to complex human diseases. Nat Genet.

[30] Ishigaki K, Akiyama M, Kanai M, Takahashi A, Kawakami E, Sugishita H (2020). Large-scale genome-wide association study in a Japanese population identifies novel susceptibility loci across different diseases. Nat Genet.

[31] Reich D, Green RE, Kircher M, Krause J, Patterson N, Durand EY (2010). Genetic history of an archaic hominin group from Denisova Cave in Siberia. Nature.

[32] Bycroft C, Freeman C, Petkova D, Band G, Elliott LT, Sharp K (2018). The UK Biobank resource with deep phenotyping and genomic data. Nature.

[33] Lek M, Karczewski KJ, Minikel EV, Samocha KE, Banks E, Fennell T (2016). Analysis of protein-coding genetic variation in 60,706 humans. Nature.

[34] Yao Y, Yang J, Xie Y, Liao H, Yang B, Xu Q (2020). No evidence for widespread positive selection signatures in common risk alleles associated with Schizophrenia. Schizophrenia Bull.

[35] Sabeti PC, Varilly P, Fry B, Lohmueller J, Hostetter E, Cotsapas C (2007). Genome-wide detection and characterization of positive selection in human populations. Nature.

[36] Abidov A, Rozanski A, Hachamovitch R, Hayes SW, Aboul-Enein F, Cohen I (2005). Prognostic significance of dyspnea in patients referred for cardiac stress testing. N Engl J Med.

[37] Ramos Rego I, Santos Cruz B, Ambrósio AF, Alves CH (2021). TRAP1 in oxidative stress and neurodegeneration. Antioxidants (Basel).

[38] Fry A, Littlejohns TJ, Sudlow C, Doherty N, Adamska L, Sprosen T (2017). Comparison of sociodemographic and health-related characteristics of UK biobank participants with those of the general population. Am J Epidemiol.

[39] Kho AN, Pacheco JA, Peissig PL, Rasmussen L, Newton KM, Weston N (2011). Electronic medical records for genetic research: results of the eMERGE consortium. Sci Transl Med.

[40] McArthur E, Rinker DC, Capra JA (2021). Quantifying the contribution of Neanderthal introgression to the heritability of complex traits. Nat Commun.

[41] Bradshaw JL, Rogers LJ (1993). The evolution of lateral asymmetries, language, tool use, and intellect.

[42] Volpato V, Macchiarelli R, Guatelli-Steinberg D, Fiore I, Bondioli L, Frayer DW (2012). Hand to mouth in a Neandertal: right-handedness in Regourdou 1. Plos One.

[43] Folkerd EJ, Dowsett M (2010). Influence of sex hormones on cancer progression. J Clin Oncol.

[44] Pardiñas AF, Holmans P, Pocklington AJ, Escott-Price V, Ripke S, Carrera N (2018). Common schizophrenia alleles are enriched in mutation-intolerant genes and in regions under strong background selection. Nat Genet.

[45] Phung TN, Huber CD, Lohmueller KE (2016). Determining the effect of natural selection on linked neutral divergence across species. Plos Genet.

[46] O’Connor LJ, Schoech AP, Hormozdiari F, Gazal S, Patterson N, Price AL (2019). Extreme polygenicity of complex traits is explained by negative selection. Am J Hum Genet.

[47] Quach H, Barreiro LB, Laval G, Zidane N, Patin E, Kidd KK (2009). Signatures of purifying and local positive selection in human miRNAs. Am J Hum Genet.

[48] Lopez-Valenzuela M, Ramirez O, Rosas A, Garcia-Vargas S, de la Rasilla M, Lalueza-Fox C (2012). An Ancestral miR-1304 Allele Present in Neanderthals regulates genes involved in Enamel formation and could explain dental differences with modern humans. Mol Biol Evol.

[49] Sirugo G, Williams SM, Tishkoff SA (2019). The missing diversity in human genetic studies. Cell.

[50] Pan-UKB team (2020). https://pan.ukbb.broadinstitute.org.

[51] Browning SR, Browning BL, Zhou Y, Tucci S, Akey JM (2018). Analysis of human sequence data reveals two pulses of archaic Denisovan admixture. Cell.

[52] Browning S (2018). Sprime results for 1000 Genomes non-African populations and SGDP Papuans.

[53] Durvasula A, Sankararaman S (2019). A statistical model for reference-free inference of archaic local ancestry. Plos Genet.

[54] Grossman SR, Andersen KG, Shlyakhter I, Tabrizi S, Winnicki S, Yen A (2013). Identifying recent adaptations in large-scale genomic data. Cell.

[55] Huber CD, DeGiorgio M, Hellmann I, Nielsen R (2016). Detecting recent selective sweeps while controlling for mutation rate and background selection. Mol Ecol.

[56] Voight BF, Kudaravalli S, Wen X, Pritchard JK (2006). A map of recent positive selection in the human genome. Plos Biol.

[57] Willer CJ, Li Y, Abecasis GR (2010). METAL: fast and efficient meta-analysis of genomewide association scans. Bioinformatics.

[58] Auton A, Brooks LD, Durbin RM, Garrison EP, Kang HM, 1000 Genomes Project Consortium (2015). A global reference for human genetic variation. Nature..

[59] Benjamini Y, Hochberg Y (1995). Controlling the false discovery rate: a practical and powerful approach to multiple testing. J R Stat Soc Series B (Methodological).

[60] Watanabe K, Stringer S, Frei O, Umićević Mirkov M, de Leeuw C, Polderman TJC (2019). A global overview of pleiotropy and genetic architecture in complex traits. Nat Genet.

[61] Chang CC, Chow CC, Tellier LC, Vattikuti S, Purcell SM, Lee JJ (2015). Second-generation PLINK: rising to the challenge of larger and richer datasets. Gigascience.

[62] Oscanoa J, Sivapalan L, Gadaleta E, Dayem Ullah AZ, Lemoine NR, Chelala C (2020). SNPnexus: a web server for functional annotation of human genome sequence variation (2020 update). Nucleic Acids Res.

[63] Ge SX, Jung D, Yao R (2020). ShinyGO: a graphical gene-set enrichment tool for animals and plants. Bioinformatics.

[64] Aken BL, Achuthan P, Akanni W, Amode MR, Bernsdorff F, Bhai J (2017). Ensembl 2017. Nucleic Acids Res.

[65] Gene Ontology Consortium (2021). The Gene Ontology resource: enriching a GOld mine. Nucleic Acids Res.

